# Assessing the effects of botulinum toxin therapy for spasmodic dysphonia: An Austria-Germany registry

**DOI:** 10.1007/s00405-025-09995-5

**Published:** 2026-02-01

**Authors:** Berit Schneider-Stickler, Gerd Fabian Volk, Oliver Galvan, Annabella Kurz, Matthias Leonhard, Tadeus Nawka

**Affiliations:** 1 Department of Otorhinolaryngology, Division of Phoniatrics-Logopedics, Medical University of Vienna, Waehringer Guertel 18-20, A-1090 Vienna, Austria; 2 Department of Otorhinolaryngology, Facial-Nerve-Center, Center for Rare Diseases, Jena University Hospital, Jena, Germany; 3 University Hospital for Hearing, Voice and Speech Disorders, Medical University of Innsbruck, Innsbruck, Austria; 4 Department of Audiology and Phoniatrics, Charité – Universitaetsmedizin Berlin, Berlin, Germany

**Keywords:** Laryngeal dystonia, Spasm count, Voice strain, Laryngeal dystonia, Dysphonia, Chemodenervation

## Abstract

**Objective:**

Injection of Botulinum neurotoxin (BoNT) is regarded as standard treatment for spasmodic dysphonia (SD), reducing the overactivity of the affected muscles. Due to the lack of standardized outcome parameters for diagnosing SD or assessing its treatment over time, the evaluation of systematic clinical evidence on the effects of BoNT therapy on SD symptom control is difficult. The registry presented in this article aimed to evaluate outcomes after BoNT treatment in SD patients in Austria and Germany, based on selected subjective and objective voice parameters.

**Methods:**

41 patients with SD were included in this multicentric registry, after drop-out of 2 patients the results of 39/41 (95.1%) patients could be analyzed per protocol. Demographic and treatment characteristics as well as the occurrence of therapy-related side effects were recorded. Perceptual voice sound evaluation (RBH scale), voice range profile measurements (VRP), number of spasms, and severity of voice strain were assessed. Patients were asked to complete the Voice Handicap Index (VHI-9i), the Communicative Participation Item Bank (CPIB), and indicate their perceived phonation effort on a VAS scale. The parameters were compared between baseline and 1-month post-BoNT treatment.

**Results:**

The patients received all BoNT type A (Xeomin^®^/Merz, Allergan^®^/Allergan or Dysport^®^/Ipsen). SD symptoms did not affect the frequency or dynamic range of the singing voice nor did they affect MPT; in contrast all the other parameters assessed were aberrant at baseline. BoNT treatment improved Jitter-%, Dysphonia Severity Index (DSI), degrees of roughness and hoarseness, but failed to restore them to normal. BoNT significantly reduced the voice spasm and strain. Phonation effort improved by approximately 50%. VHI-9i classification was reduced from moderate to mild after treatment, and CPIB from moderate to mild. The VHI-9i results were significantly positively correlated with the CPIB results.

**Conclusion:**

The results of this registry showed that BoNT injection, the current off-label treatment of SD symptoms, failed to restore normal voice quality. This registry’s outcomes also indicate that the choice of the applied BoNT brand is site-specific and does not appear to be associated with differences in efficacy. We also showed that the effects of the BoNT on the SD symptoms are best described by semi-quantitative outcome measures such as spasm counts and voice strain, and by patients-reported outcome measures (PROMs), such as CPIB and VHI-9i than by more objective parameters such as frequency and dynamic range of the singing voice.

## Introduction

 Spasmodic dysphonia (SD) is a rare focal laryngeal dystonia of unclear genesis characterized by involuntary muscle overactivity resulting in either involuntary voice breaks (adductor spasmodic dysphonia = AdSD) or a breathy voice (abductor spasmodic dysphonia = AbSD) [1–4]. Its diagnosis involves a team-approach, and it is ultimately a matter of expert opinion based on information gathered from the patient’s history and physical examination, such as the presence of typical spasmodic dysphonia signs and symptoms in the absence of comorbidities, that could account for similar problems. Due to its rarity, the diagnostic difficulties, and the lack of systematic evidence about its pathophysiology, it can be mistaken for other voice disorders, such as muscle tension dysphonia. In addition, its low prevalence prevents the medical community from developing a standardized therapy, which currently is most represented by off-label Botulinum toxin (BoNT) injection directly into the affected laryngeal muscles [1, 5]. BoNT is a neurotoxin derived from the bacteria *Clostridium botulinum*, which blocks the release of acetylcholine, responsible for the activation of muscle contractions. BoNT can be injected directly into muscles to block nerve signals, and to reduce the number and severity of spasms. The BoNT injection may decrease the number of local muscle fibre activations, thereby reducing the vocal fold adduction [6]. It has been suggested that BoNT may have an additional central nervous system (CNS) modulating effect [3], as evidenced by normalization of aberrant CNS activities after local administration [3]. This normalization would imply that BoNT could improve the acoustic and aerodynamic measures, and the patient’s perception of SD symptoms. Although off-label, BoNT treatment is considered effective, as it relieves symptoms and improves quality of life in 91% of individuals receiving treatment [5]. Different serotypes exist, but only types A and B are commercially available. They are administered similarly, but differ in dosing, onset of action and duration of action [7, 8]. The higher the dose, the weaker the muscle becomes, and fewer spasms occur. Most physicians inject BoNT transcutaneously, and many use EMG (electromyography) equipment to help with correct targeting. Some physicians deliver BoNT using a special curved needle through the mouth, sometimes using an endoscope for visual guidance [9, 10]. However, most physicians administer BoNT in an office setting without any special preparation. Physicians may inject the affected muscles on one (unilateral) or both sides (bilateral), in one or two sessions [11]. The success of BoNT therapy depends on the characteristics of the nerve-muscle interaction, which may not be fully anticipated until after several treatment cycles. The physician consequently chooses and modifies the injection route, dose, and injection site, based on clinical experience and discussions with the patient regarding symptoms and results from previous injections [12].

In AdSD, the target muscle is often the thyroarytenoid (TA) [13], although some practitioners may also target the lateral cricoarytenoid (LCA) muscle or the ventricular muscle in the false vocal fold. These muscles support the closing of the vocal folds; thus, weakening them prevents the pressed voice associated with this form of SD. In contrast, patients with abductor SD (AbSD) typically receive injections in the posterior cricoarytenoid (PCA) muscles [14]. Weakening the opening muscles leeps the vocal folds closer together during phonation, thereby preventing the breathy voice breaks associated with this form of SD. Subjects with additional tremor may also benefit from laryngeal strap muscles injections. Under certain circumstances, other laryngeal muscles, such as the cricothyroid (CT) or the interarytenoid (IA) may also be targeted [15].

BoNT is not a solution offering long-term or permanent relief from SD. The most critical limitation of the BoNT treatment is that its effects wane over time. BoNT needs to be reinjected periodically. In very few cases, patients may develop antibodies to BoNT [16].

Alternative or co-treatments to BoNT include traditional speech therapy and surgical interventions [5]. However, a study published in 2021 by Kodama et al. found that the effects of speech therapy on AdSD symptoms were poor [17]. In contrast, Tateya et al. showed that type II thyroplasty could improve the SD symptoms by altering the cortical activation, thereby reversing dysfunctional CNS patterns induced by the onset of such a condition [4]. In 2022, Sanuki et al. [18] reported from the results obtained in the treatment of AdSD with the titanium bridges between 2002 and 2014 in 385 patients in Japan. Their results were mixed with performance varying, and several bridges were reported to have broken. Notably, this device has not been approved for the EU market. The fact that presently both speech therapy and surgical intervention effects on SD symptoms are poorly studied and thus understood, leaves the BoNT injection as the favoured treatment of this condition.

Our article aims to provide evidence on the effects of the BoNT treatment on SD symptom control using different outcome measures including semi-quantitative analysis, as well as expert- and patient-subjective assessments of the voice quality before and after the treatment.

## Compliance with ethical standards 

The data presented in this a study on human participants were collected within the multicentric Austrian-Germany real-world data registry 2018CIP018 between 2018 and 2021. The study has been approved by the institutional ethic committees in Berlin (Charité – Universitätsmedizin Berlin, Germany, EA2_176_18), Innsbruck (Medical University of Innsbruck, Austria, EK Nr. 1195/2019), Jena (Jena University Hospital, Germany, 2019-1575-AWB), and Vienna (Medical University of Vienna, Austria, EK Nr. 1818/2019).

Informed consent was obtained from all participants, in compliance with the Declaration of Helsinki as amended in Fortaleza (2013).

All co-authors declare no potential conflicts of interest.

## Methods

### Data collection

The registry collected both retrospective and prospective data. The study was carried out over 3 year observation period. This resulted in the patients with different lengths of history being observed retrospectively and prospectively for the observation period. Retrospective collection included data generated within 5 years prior to registry initiation at the respective sites. The registry design allowed for the collection of the admission diagnosis from the referring doctor, patients medical history including duration and severity of voice symptoms, previous therapies, and medical examinations. The diagnosis of SD was based on medical history, perceptual evaluation of voice sound, laryngostroboscopy, and voice range profile measurements. Each site treated the enrolled SD patients according to their routine protocol. For study purposes, patients were asked to undergo specific voice tests and complete several voice-related questionnaires at baseline, i.e., before BoNT treatment, and 1-month after injection. The Principal Investigators made sure that potential effects of previous treatments had faded completely before including patients into the registry.

All centers used the DiVAS software 2.8 (XION GmbH, Berlin, Germany) to record the patients’ voice during different exercises: the patients were asked to read 10 German sentences with a high vowel content at normal pitch and loudness. The sentences were used to assess the spams and voice strain caused by the SD. A team of experienced listeners completed both assessments. In short, spasms were counted per read vowel. Only patients with at least 10 spasms at baseline were included in the analysis. Voice strain was rated on a visual analogue scale (VAS) between 0 and 10. Only patients with at least a value of 3 on the VAS at baseline were included in the analysis. The record of the patients’ reading “Der Nordwind und die Sonne” (the Northwind and the Sun) at normal pitch and loudness was used for the auditory-perceptual voice analysis was performed by means of the RBH scale (R = roughness, B = breathiness, H = hoarseness) based on a 4-point Likert scale between 0 and 3; where 0 = normal, 1 = mild, 2 = moderate, and 3 = severe [19–21]. Finally, the voice range profile protocol was recorded for each patient, to assess the fundamental frequency (F0) and sound pressure level (SPL) range in semitones (sT) and decibel (dB), respectively; the jitter in percentage; the maximum phonation time (MPT) in seconds; and the dysphonia severity index (DSI). The F0 range was considered normal if ≥ 24 sT [21]; the SPL range if ≥ 37 dB [21]; the MPT if ≥ 15 s [20, 21]. The normal range for Jitter in percentage was between 0 and 0.6% [20] and for DSI between 1.6 and 5.0 [20]. The patients were asked to complete the self-assessed Voice Handicap Index (VHI-9i) [22], the Communicative Participation Item Bank (CPIB) [23], and to define their perceived phonation effort on a VAS scale between 0 and 10, where 0 is “no effort” and 10 is “maximum effort”. The higher the CPIB score the lesser the problem with communication. The VHI-9i was additionally classified into four grades of dysphonia: none (0–5), mild (6–13), moderate (14–22), severe (23–36) [20].

### Analyses

Data were analyzed using IBM SPSS Statistics software (Version 28; IBM, New York) for medical statistics. Descriptive statistics were used to report demographic data. Continuous data were described using the mean with standard deviation and median (min, max). Qualitative data were presented as absolute and relative frequencies. Depending on the distribution, a t-test (normal distribution) or a Wilcoxon signed rank test (non-normal distribution) was used to determine significant differences. All tests were two-sided, with alpha= 5%. Questionnaires were evaluated according to their respective scoring instructions. Spearman´s Rho-correlation was used to assess correlations between the results of the different tests and questionnaires described in this article.

## Results

### Patients

A total of 41 patients were recruited in the 4 registry sites. The results of 39/41 (95.1%) patients complied with the per protocol analysis: 24 were recruited in Berlin; 9 in Vienna; 4 in Jena; and 2 in Innsbruck). 2/41 (4.9%) patients were withdrawn because they were not compliant with the registry selection criteria as the admission diagnosis from the referring doctor could not be confirmed by the Principle Investigators. The demographic information for the patients is summarized in Table 1. 9/39 patients had suffered from depressive moods most likely due to illness-related social communication limitations. In 37/39 of the patients, the SD origin was idiopathic (95%). In 1 patient the suspected cause was psychogenic after traumatic experience (2.5%) and in 1 patient the SD occurred after bronchitis.

**Table 1:** Study population

a) Patients recruitment rate at each participating site						
Site	Retrospective	Prospective	Patients recruited	Patients withdrawn	Patients for analyses	Patients at 1^st^ follow-up
Berlin	11	14	25	1	24	18
Innsbruck	0	3	3	1	2	1
Jena	0	4	4	0	4	2
Vienna	0	9	9	0	9	8
Total	**11**	**30**	**41**	**2**	**39**	**29**
b) Demographic characteristics of the recruited patients						
			**Total **			
			**n**	**Mean ± Std Dev**	**Range (min-max)**	**Median**
All patients			39			
Age			39	61 ± 17	26–85	62
BMI			35	26 ± 4	20–39	25
Age at diagnosis			39	51 ± 18	7–85	50
SD duration (years; months)			39	9;9 ± 12	0–61;4	5
Males			9			
Age			9	50 ± 16	26–79	53
BMI			8	26 ± 5	22–39	25
Age at diagnosis			9	38 ± 13	13 - 50	44
SD duration (years; months)			9	11;10 ± 12	0;2–34;8	6
Females			30			
Age			30	64 ± 15	27–85	67
BMI			27	26 ± 4	20–34	25
Age at diagnosis			30	55 ± 17	7 - 85	56
SD duration (years; months)			30	9;1 ± 12	0–61;4	5

*BMI *Body mass index, *SD *Spasmodic dysphonia, *Std Dev *standard deviation

Table 2 demonstrates the treatment overview. Before inclusion in this registry, 34/39 (87.2%) patients had received at least one BoNT injection. Among them, 23/34 (67.6%) had undergone speech therapy in addition to the BoNT injection. Only 1/39 (2.6%) patient had undergone speech therapy only. Voice therapy was initially prescribed by practicing physicians or at the express request of the patients.

**Table 2:** Treatment overview. a) Frequencies of each SD therapy recorded at baseline b) Number of Botulinum toxin (BoNT) injections received before baseline by the patients who had undergone this treatment in the past

a)						
Treatment*	All patients (*n* = 39)		Males (*n* = 9)		Females (*n* = 30)	
	*n*	%	*n*	%	*n*	%
Speech therapy only	1	2.6%	1	11.1%	0	0.0%
BoNT injection only	10	25.6%	2	22.2%	8	26.7%
BoNT injection and physical training	1	2.6%	0	0.0%	1	3.3%
BoNT injection and speech therapy	20	51.3%	3	33.3%	17	56.7%
BoNT injection, speech therapy and psychological support	3	7.7%	2	22.2%	1	3.3%
No treatment	4	10.3%	1	11.1%	3	10.0%
b)						
Number of BoNT Injections*	**All patients** (*n*=39)		**Males** (*n*=9		** Females** (*n*=30)	
	**n**	**%**	**n**	**%**	**n**	**%**
Never	1	2.6%	1	11.1%	0	0.0%
Received once only	3	7.7%	1	11.1%	2	6.7%
Received between 2 and 5 doses	8	20.5%	1	11.1%	7	23.3%
Received more than 5 doses	23	59.0%	5	55.6%	18	60.0%
Unknown	4	10.3%	1	11.1%	3	10.0%

^*^Per protocol set at baseline

All the 39 patients included completed the baseline analysis and 29/39 patients (76.9%) completed the 1-month post-treatment follow-up. However, not all the 29 patients completed the entire series of tests foreseen for both checkpoints. Since all study centers involved are contact points for supra-regional patient referrals, it is not always possible for patients to keep to the scheduled check-up appointments due to sometimes long and arduous journeys. Thus, the number of patients on whom the results could be assessed at baseline and during the 1-month follow-up varies from test across tests.

Although the registry allowed the recruitment of patients undergoing any type of SD treatment, all the enrolled patients received a BoNT injections without speech therapy. The BoNT was administered in the thyroarytenoid muscle (TA) in 36 patients (92.3%), and in the cricothyroid muscle (CT) in 1 patient (2.6%). 2 (5.1%) patients with abductory SD were included, therefore, the BoNT was administered in the posterior cricoarytenoid muscle (PCA). The mean BoNT dose given at baseline was 3.4 ± 3.1 I.U. with a median of 2.5 I.U. AllerganÒ was used in 22/39 (56.4%); XeominÒ in 10/39 (25.6%); BotoxÒ in 5/39 (12.8%); and DysportÒ in 2/39 (5.2%) cases. The BoNT administration was always performed under LEMG guidance.

### Treatment related adverse events

None of the patients enrolled in this registry reported any treatment-related adverse events (AEs), such as swallowing difficulties, aspiration or a very over-ventilated voice sound with limitation of communicative skills.

### Number of spasm and voice strain 

As demonstrated in Table 3, spasm count was analyzed in 20/39 patients at baseline and 1 month after BoNT treatment, even though 22/39 patients completed the test at both checkpoints, since 2 cases were excluded because their baseline spasm count was <10.

**Table 3:** Spasm counts and voice strain at baseline and 1-month after BoNT injection

		Baseline	1-month	*p*-value^Η^
Spasm count	n	20	20	<0.001**
	Mean ± Std Dev	50.7 ± 30.9	9.9 ± 16.3	
	Range	10.0–98.0.0.0	0.0–57.0.0.0	
	Median	49.5	0.0	
Voice strain	n	18	18	<0.001**
	Mean ± Std Dev	7 ± 2	2 ± 2	
	Range	3.0–10.0.0.0	0.0–6.0.0.0	
	Median	7.0	2.0	

^*Η*^: Wilcoxon signed rank test; *p<0.05; **p<0.001

Voice strain was analyzed in 18/39 patients at baseline and 1 month after BoNT treatment, even though 22/39 patients completed the test at both checkpoints, since 4 cases were excluded because their baseline voice strain value was < 3.

### Auditory-perceptual voice analysis (RBH)

Table 4 shows the RBH assessment. BoNT treatment significantly improved voice roughness (p=0.047), but had no significant impact on breathiness (p=0.441) and hoarseness (p=0.067) as depicted in Table 4.

**Table 4:** Roughness (R), Breathiness (B), Hoarseness (H) assessment at baseline and 1-month after BoNT injection

		Baseline	1-month	*p*-value^Η^
R	n	24	24	0.047*
	Mean ± Std Dev	2 ± 1	1 ± 1	
	Median	2	1.	
	Mode	1	1	
B	n	24	24	0.441
	Mean ± Std Dev	1 ± 1	1 ± 1	
	Median	1	1	
	Mode	1	0	
H	n	24	24	0.067
	Mean ± Std Dev	2 ± 1	1 ± 1	
	Median	2	1	
	Mode	3	1	

*R* Roughness, *B *Breathiness, *H* Hoarseness, *Std Dev* Standard deviation. ^*Η*^:Wilcoxon signed rank test; *p<0.05; **p<0.001

### Voice range profile measurement

The results of the VRP measurement are demonstrated in Table 5. F_0_ and SPL range were found to be normally and MPT non-normally distributed, respectively. All the 3 outcome measures were found within their normal range at baseline and were not significantly affected by the BoNT treatment. In particular, the DSI calculated before and after treatment indicated a severe dysphonia.

**Table 5:** Voice range profile assessment at baseline and 1-month after BoNT injection. F0= fundamental frequency; SPL= sound pressure level; MPT=maximum phonation time; DSI= dysphonia severity index; sT=semitones; dB=decibel; s=seconds

		Baseline	1-month	p-value^¥,Η^	
F_0_ Range (sT)	n	22	22	0.174^¥^	
	Mean ± Std Dev	19.7 ± 10.0	23.0 ± 10.3		
	Median	19.1	22.8		
SPL (dB)	n	22	22	0.114^¥^	
	Mean ± Std Dev	28.3 ± 13.8	33.4 ± 12.8		
	Median	30.0	34.0		
MPT (s)	n	22	22	0.966^Η^	
	Mean ± Std Dev	11.5 ± 7.8	11.7 ± 10.0		
	Median	8.6	7.1		
Jitter (%)	n	21	21	**0.002**^Η^*	
	Mean ± Std Dev	12.9 ± 13.8	5.5 ± 7.2		
	Median	7.5	1.8		
DSI	n	21	21	**0.003**^Η^*	
	Mean ± Std Dev	−10.5 ± 13.1	−3.6 ± 10.2		
	Median	−7.8	0.2		

^*Η*^*:Wilcoxon signed rank test; *^*¥*^: Paired samples t-test; *p<0.05; **p<0.001

### VHI-9i

The results for VHI-9i are shown in Table 6. VHI-9i was normally distributed and showed a significant (p<0.001) improvement 1-month after BoNT administration.

**Table 6:** Phonation effort, voice handicap Index, and the communicative participation item bank (CPIB) in patients at baseline and 1-month after BoNT injection

	Baseline		Baseline	1-month		*p*-value^Η^
Phonation Effort	n		24	24		< 0.001**
	Mean ± Std Dev		7.5 ± 1.7	4.1 ± 2.7		
	Median		8.0	3.5		
	Range (min.-max.)		3–10	1–10		
VHI-9	n		24	24		< 0.001**
	Mean ± Std Dev		23.5 ± 6.5	14.7 ± 8.3		
	Median		22.5	12.5		
	Range (min.-max.)		13–36	2–32		
CPIB	n		23	23		< 0.001**
	Mean ± Std Dev		12.3 ± 9.2	21.0 ± 7.7		
	Median		12.0	23.0		
	Range (min.-max.)		0–28	3–30		

^*Η*^ : Wilcoxon signed rank test; *p < 0.05; **p < 0.001

### Communicative Participation Item Bank (CPIB)

CPIB was completed by 23/39 patients at baseline and 1-month after BoNT treatment. CPIB was non- normally distributed and showed a significant (p<0.001) improvement 1-month after BoNT administration, passing from an average of 12.3± 9.2 [0–28] and a median of 12, compatible with a moderate handicap, to an average of 21 ± 7.7 [3–29] and a median of 23, compatible with a mild handicap.

### Phonation effort

Phonation effort was assessed in 24 of 39 patients at baseline and 1-month after BoNT treatment. Phonation effort was non-normally distributed and showed a significant (p<0.001) improvement 1-month after BoNT administration, passing from an average of 7.5 ± 1.7 [3–10] and a median of 8, to an average of 4.1 ± 2.7 [1–10] and a median of 3.5.

### Correlation analyses

In Table 7, the correlation between spasm count, voice strain, VHI-9i, CPIB and phonation effort both at baseline and 1-month after BoNT injection is demonstrated. The results of the CPIB at baseline and 1-month after BoNT correlated with those of the VHI-9i (ρ=−0.824, p<0.001). The results of the VHI-9i, but not of CPIB, correlated significantly with the phonation effort (ρ=0.656, p=0.011) but only 1-month after BoNT. Only at this time point, the voice strain and the spasm count correlated significantly (ρ =0.804, p<0.001).

**Table 7:** Correlation between spasm count, voice strain, VHI-9, CPIB and phonation effort both at baseline and 1-month after BoNT injection

								Coef.			
Baseline			n		Mean		SD	Spasm count	Voice strain	VHI-9	CPIB
Spasm count			14		60.4		28.4	-			
Voice strain			14		7.6		1.9	0.406	-		
VHI-9			14		24.6		6.9	−0.084	−0.327	-	
CPIB			14		11.1		8.6	0.009	0.336	−0.760**	-
Phonation effort			14		7.9		1.7	−0.163	−0.009	0.381	−0.663*
a)											
Coef.: Spearman correlation coefficient; *p < 0.05 **p < 0.001											
								Coef.			
1-month			**n**		**Mean**		**SD**	**Spasm count**	**Voice strain**	**VHI-9**	**CPIB**
Spasm count			14		7.9		16.8	-			
Voice strain			14		2.1		1.9	0.804**	-		
VHI-9			14		12.9		7.8	0.509	0.235	-	
CPIB			14		21.7		7.8	−0.234	0.014	−0.824**	-
Phonation effort			14		3.4		2.8	0.317	0.446	0.656*	−0.447
b)											

Coef.: Spearman correlation coefficient; *p<0.05 **p<0.001

## Discussion

While the registry aimed to assess the effects of various therapies on SD symptoms, all recruited and analysed patients received BoNT injections. This is in line with the fact, that because of decades of experience, all involved sites consider speech therapy alone to be a poor treatment for SD symptoms.

Before inclusion in this registry, 34/39 (87.2%) patients had received at least one BoNT injection. Among them, 23/34 (67.6%) had undergone speech therapy in addition to the BoNT injection. Among those who had received at least one BoNT injection before enrolment, 31/34 (91.2%) had undergone the treatment at least twice (see Table2 for details). It was therefore not possible to investigate in this patient cohort whether the effect of initial treatment with BoNT differs from the effects of subsequent injections.

As reported in the literature [24], the effects of BoNT injection on SD symptoms are expected to reach their peak effect within 7 days and gradually decrease within 13.6 weeks. Accordingly, based on the medical history of the patients recruited into the registry, most (70.6%) got a repeated treatment every 3–6 months. Thus, the endpoint at 1-month after BoNT injection should allow the assessment of the treatment’s effectiveness at its peak. In 2017, Namin et al. [25] reported that the average BoNT dose inpatients with AdSD deceased significantly during the initial 10–15 cycles and stabilized thereafter. In the registry, most of the patients (59%) had undergone more than 5 cycles before enrolment, and 20.5% had received 3–4 cycles at enrolment. Based on these premises, the population analysed in this registry is relatively homogeneous and it is reasonable to expect that the observed BoNT effects could be generalized and showed a low individual-characteristic bias, which was further reduced by the fact that, as expected [7, 26], 76.9% of the population was female. Interestingly the average age of the female population was about 14 years older than that of the male population, and the initial diagnosis was performed about 17 years later than in the males (see Table 1b for details).

In this registry, all BoNT brands available in the EU were used, with a site preference for each brand. The use of different brands may indicate that brand choice is subjective and not linked to objective differences in their efficacy. As expected [27], the TA muscle (94.7%) was the preferred target for AdSD treatment, and the injection was bilateral. The mean BoNT dose administered in this registry was 3.4 ± 3.1 I.U. (range = 1–15] with a median of 2.5 I.U., which was almost twice the dose published by French et al. in 2020 [28], and nearly half the dose published in [27]. This result further highlights the lack of standardization in the method, which is strongly dependent on the administration protocol and the availability of guidance, such as EMG for the selective injection. Interestingly, when the dosage in this registry was compared with that used in another study in which BoNT was injected under EMG guidance [29], the latter study’s reported average dose was exactly the median dose in this registry. Over the last years, the assessment of the SD symptoms and treatment effects has become more matter of debate in the scientific and medical communities. As reported in 2022 by Rumbach et al. [30], there is no consensus on the outcomes to be used to diagnose SD and assess the relevant treatment effectiveness. Patient-reported outcomes such as CPIB [23], and spasm counts [31], are promising outcome measures that may improve the assessment of SD symptoms and treatment. However, their use is far from being routine and, regarding spasm count, there is a lack of a standardized protocols that may significantly bias the results and reduce the comparability among studies conducted by different groups in different countries and using different languages. In this registry, we assessed the most common outcomes measures for the assessment of voice disorders along with the spasmodic dysphonia specific parameters such as spasm count, voice strain, and phonation efforts, which may still require additional standardization to become routine. The results showed that the most objective outcome measures such as frequency and dynamic ranges, and MPT were not or only subclinically affected by SD, unlike less standardized parameters such as Jitter and DSI, which were relevantly worsened by SD. As expected, spasm count, voice strain, and phonation efforts were strongly affected by SD as well as voice roughness and hoarseness but not breathiness. The patients themselves rated their voice quality as severely affected and their communication ability as moderately affected by SD. Based on these premises, it seems that while objective voice quality measures do not detect clinically relevant worsening of the voice, both expert voice therapists and patients perceived a moderate to severe voice deterioration that a standard voice diagnostics software cannot quantify. Interestingly, while BoNT therapy significantly improved all the aberrant parameters described above, it failed to improve a normal voice quality which changed from severe to moderate or from moderate to mild. Because of the subjective components of all the aforementioned tests and questionnaires, the results may have been biased by the operator or the patient. For instance, patients’ perception but not those of experts may have been biased by psychological conditions such as stress and depression, which are often associated with SD [32]. On the other hand, the lack of standardized methods for assessing spasm count and voice strain may have affected the test results. The most apparent effect of the BoNT appears to be a significant reduction in both the number of spams and voice strain. However, this effect does not lead to normalization of perceived voice quality by the patients (VHI-9i and phonation effort) or by the experienced phoniatricians (roughness and hoarseness). Regarding the 2 voice questionnaires, it was interesting to observe that that, while the patients described their voice quality as severely affected by SD, they reported their communication problems as only moderate. The BoNT reduced communication issues to a mild level, while the problem with voice quality remained moderate. n issues to a mild level, while the voice quality problem remained rated as moderate.

The small sample size did not allow to stratify the groups of dystonia (ABSD, ADSD, and dystonic tremor). The most critical limitation of this registry is the small sample size and the fact that, as an example of RWD collection, not all patients completed all tests and questionnaires. Patient recruitment was highly dependent on hospital size, with 89.7% of the patients recruited in the two larger hospitals. Still, the similarity among the patients recruited across the various sites regarding treatment, SD symptoms, and demographics should have relevantly reduced the potential biases. The gender, age, and BMI of the recruited patients also showed no relevant study-specific correlation. The spasm number, voice strain, and RBH assessment were performed by the same team on anonymized datasets, and none of the team members participated in the recruitment.

## Conclusions

The results of this registry showed that, most of the patients received frequent BoNT injections. The choice of the applied brand is site-specific and does not appear to be associated with differences in efficacy. We also showed that the effects of BoNT on SD symptoms are best described by semi-quantitative outcome measures, such as spasm counts and voice strain, and by patients’ reported outcome measures (PROMs), such as CPIB and VHI-9i. More objective parameters, such as frequency and dynamic range of the singing voice, do not appear to change significantly because of treatment for this disease. These findings emphasize the need for more systematic evaluation and standardization of outcome measures for both the diagnosis and treatment of SD. Based on this, increased collaboration among hospitals can yield clinical insights that improve patient care. 
